# *FolE* gene expression for folic acid productivity from optimized and characterized probiotic *Lactobacillus delbrueckii*

**DOI:** 10.1186/s43141-023-00603-9

**Published:** 2023-12-18

**Authors:** Mohamed Khedr, Fady Sayed Youssef, Noura El-kattan, Mahmoud S. Abozahra, Mohammed N. Selim, Abdullah Yousef, Kamal M. A. Khalil, Alsayed E. Mekky

**Affiliations:** 1 Department of Botany and Microbiology, Faculty of Science, Al-Azhar University, Nasr, 11884 Cairo Egypt; 2 Department of Pharmacology Faculty of Veterinary Medicine, Cairo University, Giza, 1221 Egypt; 3 Department of Microbiology, Research Institute of Medical Entomology, General Organization for Teaching Hospitals and Institutes, Giza, Egypt; 4 Department of Chemistry and Biochemistry, Florida Atlantic University, Boca Raton, FL 33433 USA; 5 Microbial Chemistry Department, National Research Centre, 33 El-Buhouth Street, Dokki, 12622 Cairo Egypt; 6 Basic & Medical Sciences Department, Faculty of Dentistry, Alryada University for Science & Technology, Al ryada, Egypt; 7 Genetic Engineering and Biotechnology Division, Genetics and Cytology Department, National Research Centre, 33 El-Buhouth Street, Dokki, 12622 Cairo Egypt

**Keywords:** *Lactobacillus delbrueckii*, Folic acid, *FolE* gene, DHFR, Antioxidant

## Abstract

**Background:**

*Lactobacillus delbrueckii* was one of the most common milk lactic acid bacterial strains (LAB) which characterized as probiotic with many health influencing properties.

**Results:**

Among seven isolates, KH1 isolate was the best producer of folic acid with 100 µg/ml after 48 h of incubation; *FolE* gene expression after 24 h of incubation was in the highest value in case of KH1 with three folds. Lactose was the best carbon source for this KH1, besides the best next isolates KH80 and KH98. The selected three LAB isolates were identified through 16S rDNA as *Lactobacillus delbrueckii*. These three isolates have high tolerance against acidic pH 2–3; they give 45, 10, and 22 CFUs at pH 3, besides 9, 6, and 4 CFUs at pH2, respectively. They also have resistance against elevated bile salt range 0.1–0.4%. KH1 recorded 99% scavenging against 97.3% 1000 µg/ml ascorbic acid. Docking study exhibits the binding mode of folic acid which exhibited an energy binding of − 8.65 kcal/mol against DHFR. Folic acid formed four Pi-alkyl, Pi-Pi, and Pi-sigma interactions with Ala9, Ile7, Phe34, and Ile60. Additionally, folic acid interacted with Glu30 and Asn64 by three hydrogen bonds with 1.77, 1.76, and 1.96 Å.

**Conclusion:**

*LAB isolates* have probiotic properties, antioxidant activity, and desired organic natural source for folic acid supplementation that improve hemoglobin that indicated by docking study interaction.

**Graphical Abstract:**

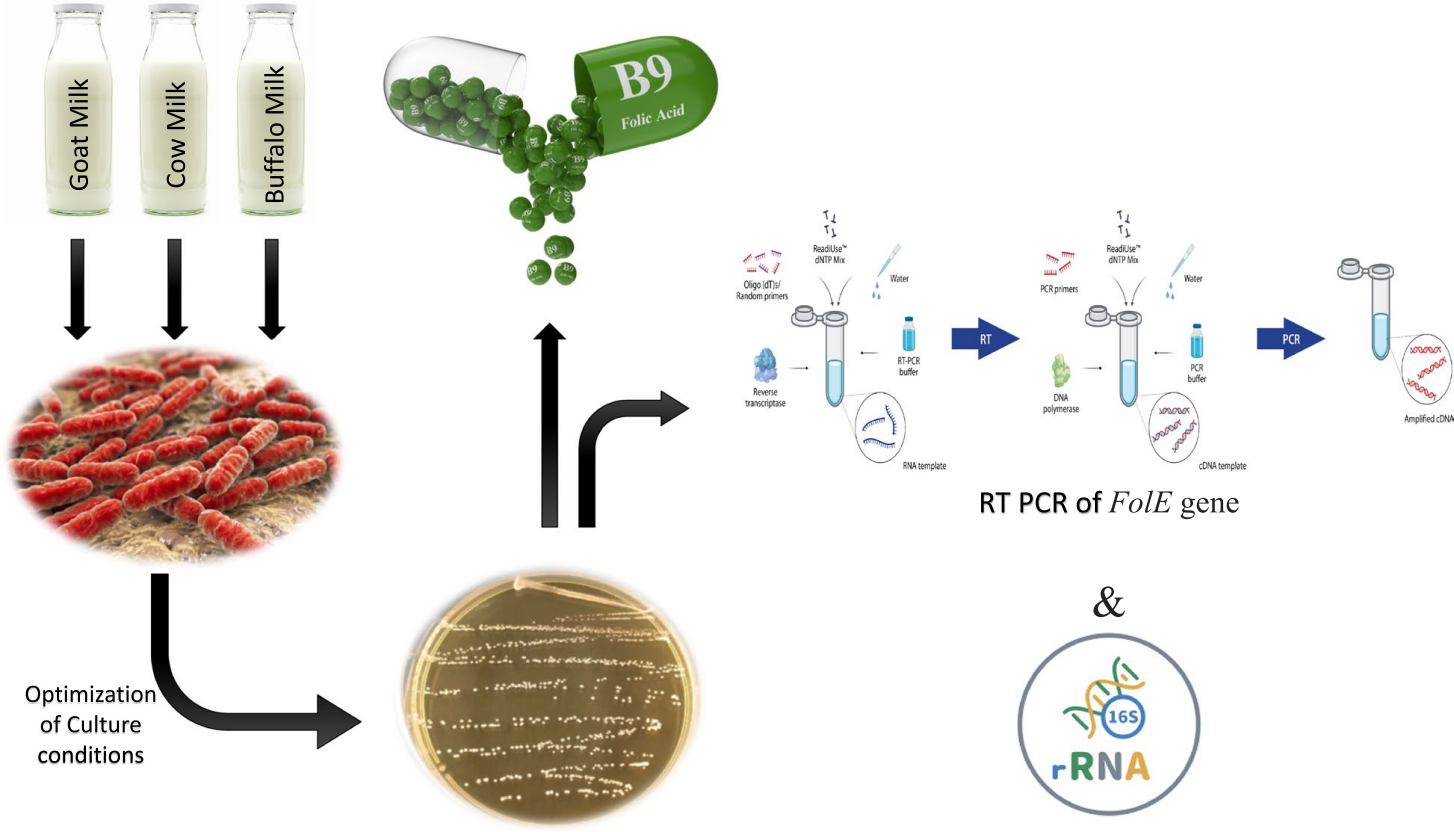

## Background

*Lactobacilli* were mostly used bacterial strains that have numerous applications in industry and human health field like probiotics and food preservation [1]. Most probiotic microorganisms belong to the lactic acid bacteria (LAB) group. LAB exhibits fermentation activities which were exploited in food preservation for previous many years. *Lactobacilli* are the most famous group of (LABS) that are used as probiotics [2].* Lactobacillus* strains have remarkable health-promoting and preservation activities [3].

LAB is used as starter to improve the texture, flavor, and nutritional value of some products, such as sourdough, cheese, silage, beer, wine, fermented plant, and meat [4–6]. Specific probiotic strains have positive effects on atopic eczema [7], irritable bowel syndrome [8], diarrhea [9], and antibiotic-related diarrhea [10, 11]. Many *Lactobacillus* strains have strong antimicrobial efficiency with remarkable inhibitory activity against *Salmonella* [12, 13].

Probiotics live in the gut and bind with epithelial cells like *Lactobacillus* and *Bifidobacterium* and yeast like *Saccharomyces cerevisiae* to prevent pathogen replacement and play an important role in health [14, 15]. It rapidly colonizes the intestinal epithelium by the genus* Lactobacillus*, produces bacteriocin and lactic acid, and lowers the pH, thus impeding the growth and reproduction of intestinal pathogens.

Any folic acid shortage in the body of a person results in severe anemia symptoms. Because it is a rate-limiting enzyme, dihydrofolate reductase (DHFR) has an advantage in the activation of folic acid. To show the degree of affinity towards this target, we aim to present the interaction between folic acid and DHFR target sites in the current work. The molecular docking procedure was carried out using the molecular operating environment (MOE) program [16].

In addition, *Lactobacillus* increases macrophage activity and production of immunoglobulin IgA, which plays an important role in the immune system, such as local regulation of immune responses, allergies, and inflammatory diseases [17–21]. This study was conducted to identify and characterize the most potent LAB isolates as an alternative and safe source of folic acid supplementation besides other benefits like maintain gut normal flora in human that improve general body health.

## Methods

### Bacterial isolation

Twenty-five isolates from different milk samples were examined morphologically through Gram stain reaction, M17 and MRS agar, then maintained in MRS broth at 4°C. Then they used to test their antimicrobial activity against pathogenic multi-drug resistant strains.

### Standard strain

*Lactobacillus delbrueckii* ATCC 730, which was glycerol-maintained strain, was sub-cultured and activated on MRS broth at 30°C for 48 h within ISS-4075/ISS-4075R Incubated Shaker with speed 100 r.p.m.

### Folic acid assay

#### Preparation of folic acid standard solution

**Fig. 1:**
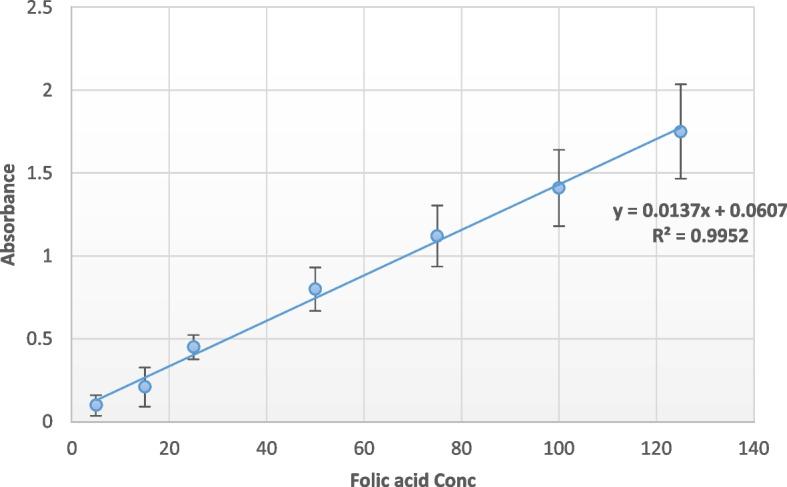
Folic acid standard linear according to absorbance values at 285 wavelength for known concentrations from 5 to 125 µg/ml

Individual folate standards, tetrahydrofolate (THF), 5-methyltetrahydrofolate (5-MeTHF), and 5-formyltetrahydrofolate (5-FmTHF) were further purified and prepared [22]. Standard linear values of folic acid were compared with samples through UV spectrophotometer at 285 nm wavelength (Fig. 1).

### Extraction and deconjugation of folic acid

Six milliliters of culture was added to 10 ml of extraction buffer (0.1 M phosphate buffer includes 0.5% sodium ascorbate). The mixture is then located within a boiling water bath for 15 min followed by centrifugation at 4000 × *g* for 10 min. About 0.4 ml of human plasma was added to 3 ml of obtained supernatant. Human plasma contains deconjugatives that deconjugate the polyglutamine of folic acid to the mono glutamine form. The mixture was incubated for 1 h at 37°C with continuous rotation. The reaction was terminated by placing the sample in boiling water bath for 5 min. Extracts were centrifuged at 27,000 × *g* for 10 min. The supernatant was then filtered through 0.45-µm filter and either used directly or stored at − 20 °C until use [22].

### Ribonucleic acid isolation and complementary deoxyribonucleic acid synthesis

#### Ribonucleic acid isolation

Four total ribonucleic acids **(**RNAs) were isolated from 72 h LAB strains in liquid MRS medium, using Trizol (Gibcol) according to the manufacturer’s protocol. First strand cDNA was synthesized using Advantage RT-for-PCR Kit (Clontech, Palo Alto, CA, USA).

#### Conventional reverse transcription polymerase chain reaction amplification

Polymerase chain reaction (PCR) was performed in 50 μl reactions using primers (Table 1) from [23]. A PCR reaction consists of 100 ng of synthesized cDNA, 200 mM dNTPs, 0.1 mM of each primer, 1.5 mM MgCl2, and 1 U Taq DNA polymerase (Takara), and sterile water to bring the volume to 50 μl. This is the PCR program:

**Table 1:** Specific primers are used to detect *FolE* and *RecA* genes in three LAB strains and standard one

Primers	Sequence (5'- > 3')	Template strand	Length	Tm	GC%
FW 1FolE	GCCAGCATAGTCTGAAGTGT	Plus	20	57.61	50.00
RW 1 FolE	GCAAAAAGCCAACGATGCAAG	Minus	21	59.81	47.62
FW 2 FolE	TCAACTAGCCGCGGAATTTT	Plus	20	58.18	45.00
RW 2 FolE	TGGCAGTAGGTGAAGATCCTG	Minus	21	59.17	52.38
FW 1RecA	GCCCTAATTGGTCCAGGCG	Plus	19	44.3	44.3
RW 1 RecA	ACAACGGCGTTCTCTCCTAT	Minus	20	44.3	44.4
FW 2 RecA	ACACAACGTCATTGCAAATGTGA	Plus	23	44.3	44.3
RW 2 RecA	GCCTGGACCAATTAGGGCAT	Minus	20	44.3	44.3

Denaturation at 94°C for 4 min, 30 cycles of 94°C for 30 s, 56°C for 50 s, 72°C for 1 min, and final extension at 72°C for 6 min. Agarose gel separated in % TAE buffer (40 mM Tris–Acetate, pH 7.6- and 1-mM Na-acetic acid (EDTA)). After electrophoresis, gels were stained with ethidium bromide (0.5 mg/ml) and visualized with UV light. Sizes were estimated by comparison with standard-length DNA (GeneRuler™ 100 bp DNA Ladder, MBI Fermentans, Vilnius, Lithuania).

#### Real time PCR amplification conditions

Complementary DNA (cDNA) from four samples was semi-quantitatively PCR using the primers shown in Table 1. The first four primers are specific to its *FolE* gene, and the last four primers are specific to its *RecA* gene, housekeeping gene. The real-time PCR reaction consisted of 12.5 μl of 2 × Quantitech SYBR® Green RT mix (Fermentase com.), 1 μl 25 pm/μl forward primer, 1 μl 25 pm/μl reverse primer, 1 μl cDNA (50 ng), and 9.25 μl of RNase-free water for a total of 25 μl. Samples were centrifuged before being loaded into the rotor wells. The real-time PCR programs are as follows:

Initial denaturation at 95°C for 10 min; 40 cycles of 15 s at 95°C. Incubate for 30 s at 59°C and extend for 30 s at 72°C. Data collection is done in the expansion phase. Reactions were performed using a Rotor Gene 6000 system “Qiagen, USA”. The 18S ribosomal RNA (rRNA) gene was used as a housekeeping gene (reference gene) in this trial. The cutoff cycle (CT) values represent the PCR cycle at which the first increase of fluorescence above a defined threshold occurred in each gain curve.

### pH tolerance test

Isolates were inoculated in the brain heart fusion broth at optimized sugar source concentration for each isolate besides other nutritional factors which tested previously. Serial dilution was performed and about 100 µl of fermentation after 24 h of incubation was spread via glass rod on MRS agar and incubated at an appropriate CO_2_ concentration for 24 h.

### Bile salt tolerance

Seven isolates were tested for their tolerance to different concentrations of chenodeoxycholate as bile salt. Firstly, isolates were selected as CFUs from MRS agar, then re-suspended in MRS with the adjusted pH for each isolate and bile salt at the ratios 0.1 to 0.5%, and then inoculated on MRS agar; CFU/mL cells were counted.

### Molecular identification of Lactobacilli

PCRs were carried out in a programmable DNA thermal cycler PCR system. PCR reaction mixture was optimized to 25 μl that contains 6 μl of template (bacterial) DNA solution and 8.5 μl of master mix, which includes dNTPs mix, MgCl_2_, Taq polymerase, and PCR buffer.

Primers were added separately after preparation from lyophilized stock (1 μmol/l of each primer). Specific two primers were listed in Table 2. The amplified DNA product along with a DNA marker gene ruler 100 bp DNA ladder Ayonex was separated by electrophoresis using 1.5% agarose gel within TAE. The gel was stained with ethidium bromide, and the banding profile was recorded using UV-gel documentation system. The amplified DNA product was purified and sequenced.

**Table 2:** Two PCR universal bacterial primers used for 16s rDNA amplification of *lactobacilli* isolates

Primers	Sequence
FW-Khedr16bac	5-GATCCTGGCTCAGACGAACG-3
RW-Khedr16bac	5-GCTACGCATCATTGCCTTGG-3

### Total antioxidant capacity

(TAC)^+^Radical mono cation of (2,2’Azinobis (3-ethylbenzothiazoline-6 sulphonic acid)). By oxidizing ABTS with potassium persulfate and reducing it in the presence of antioxidants that donate hydrogen, ABTS was created, which was then detected using a UV1902 spectrophotometer at 734 nm. The total antioxidant capacity was evaluated using this decolorization assay [24]. The concentration of antioxidant and duration of reaction on the inhibition of the radical cation absorption were considered when the antioxidant activity was determined. Trolox, a water-soluble analog of vitamin E, was used as a positive control. The activity was expressed in terms of Trolox equivalent antioxidant capacity (TEAC)/mg extract.

### Method of docking study

Folic acid has a main role in RBCs development and maturation. In normal physiological erythropoiesis, folic acid was converted to deferent derivatives to be ready for forming RBCs. Any deficiency in folic acid concentrations in the human body leads to serious manifestation of anemia. DHFR is a rate limiting enzyme; it has upper hand in the activation of folic acid. In the present study, we try to present the interaction of folic acid and DHFR target sites to show the strength of affinity towards this target. The molecular docking process *was done* by using molecular operating environment (MOE) software. Crystal protein was obtained from protein data bank (PDB codes: 1DRF).

#### Preparation of targeted proteins

Protein structure was obtained from protein data bank; at first, water molecules were excluded from the complex. Then, quick preparation was done, missing amino acids were added, and unfilled valence atoms were corrected. Protein peptide energy was minimized by applying CHARMM force fields. The protein essential amino acids were selected and prepared for screening.

#### Preparation of the tested candidates

2D structure of folic acid was drawn using Chem-Bio Draw Ultra17.0 and saved in SDF file format. From MOE software, the saved file was opened, ligand was protonated, and energy was minimized by applying 0.1 RMSD kcal/mol, a MMFF94 force field.

### Data analysis

- RT-PCR: Comparative quantification analysis was done using dd∆ct method through Microsoft Excel.

- Standard deviation and standard error statistics were evaluated through ANOVA incorporated Microsoft Excel.

## Results

### Lactobacilli isolation and cultivation

**Fig. 2:**
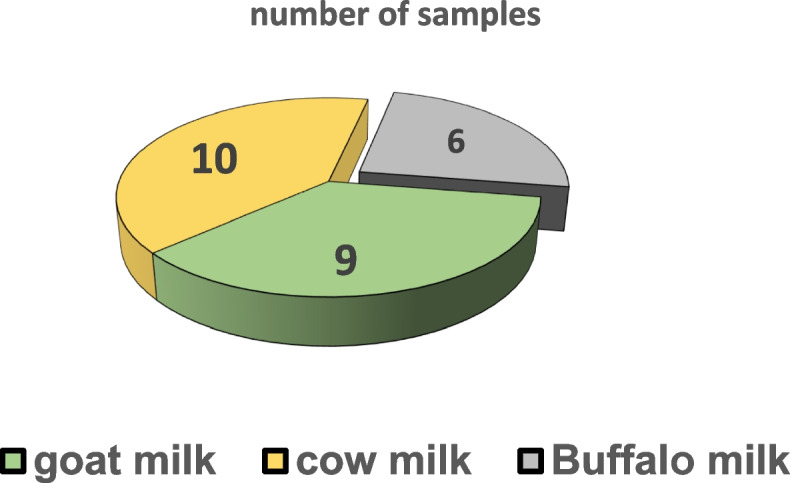
Number of isolated milk samples from different three sources, cow, goat, and buffalo

To obtain probiotic bacteria, LAB isolates were obtained from Egyptian traditional fermented items based on the most relevant scientific, functional, and health criteria. Based on their morphological traits and physiological and biochemical properties, among 25 bacterial isolates were collected from 25 different milk samples through serial dilution method, 10 samples from goat milk, 9 from cow milk, and 6 from buffalo milk (Fig. 2).

One hundred colonies include white color with rounded edges and appeared as microscopic dots on MRS agar. Rogosa agar was used as a selective and confirmed step for *Lactobacilli* isolates. Each serial dilution factor was poured in three MRS agar (triplicated), and plates were incubated for 24 h at 37°C and CO_2_ concentration (5.5%). After incubation, CFUs were counted and examined for their gram stain reaction, and morphological examination of colonies; about 100 hundred CFUs reacted positively with gram reaction and appeared bacillary form under light microscope.

### Carbon source optimization

Seven isolates among 25 isolates were selected according to their ordinary cultivation temperature at 30°C, besides their rapid and ideal growth on MRS and Rogosa agar, cultivated on MRS broth. These seven isolates were tested on different carbon sources (glucose, mannose, fructose, sucrose, and lactose), with constant other factors like temperature at 30°C and CO_2_ concentration (5.5%), shaking at 150 r.p.m. for 3 days; within 3 days after each 6 h, samples were collected from each isolate and measured for cell growth turbidity at OD600 through UV spectrophotometry.

**Fig. 3:**
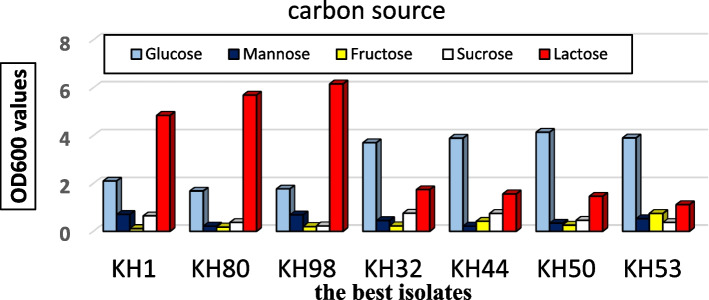
Colorimetric growth of the best seven isolates which gave maximum growth with lactose (the first three from left) and with glucose (the last four)

Almost all of the samples were grown exponentially against control samples (without carbon source); in the case of lactose and glucose as a carbon source, four isolates which gave maximum growth with lactose were KH32, KH44, KH50, and KH53, while another three reached maximal values with glucose were KH1, KH80, and KH98 (Fig. 3).

### Folic acid production

**Fig. 4:**
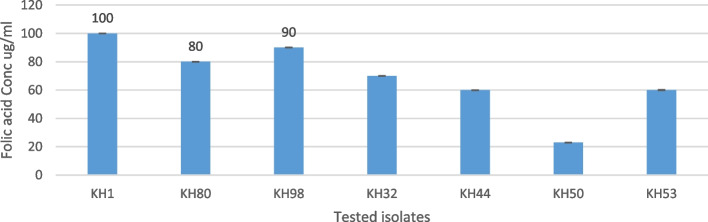
Folate production of the best seven LAB isolates after 48 h of incubation in MRS broth

Seven isolates were evaluated for their folic acid production. It measured through colorimetric assay against folic acid standard with known values as in Fig. 1. The maximum folate productivity achieved in KH1 isolate was 100 µg/ml after 48 h of incubation, while after the same incubation time, KH98 recorded productivity of 90 µg/ml followed by KH80 with 80 µg/ml. The rest of the isolates showed lower productivity than the three mentioned highest productivity (Fig. 4).

### FolE gene expression

**Fig. 5:**
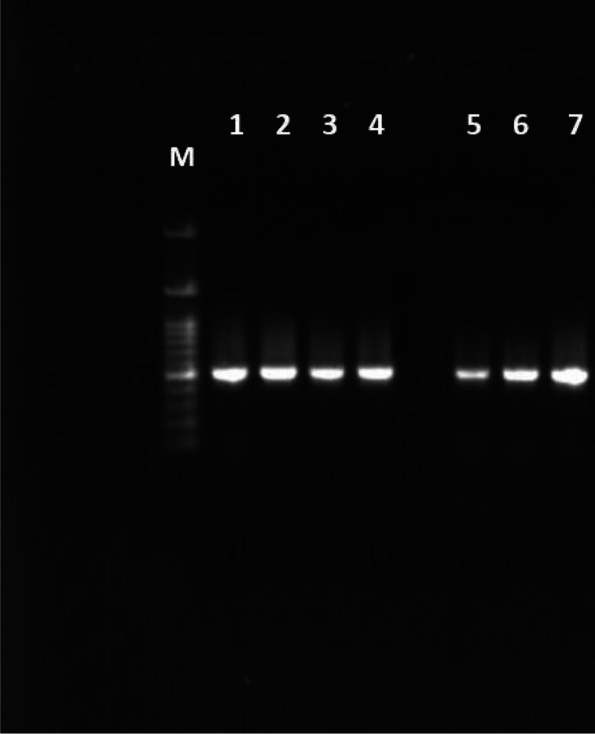
Agarose gel electrophoresis of FolE gene amplicon with DNA ladder marker (Band M), KH1 (Band 1), KH80 (Band 2), KH98 (Band 3), KH32 (Band 4), KH44 (Band 5), KH50 (Band 6), and KH53 (Band 7)

PCR detection and amplification of *FolE* gene in one standard strain and the seven isolates were done where the amplicons with molecular weight have 1470 basis points (bps) in size in agarose gel electrophoresis against wide range DNA ladder with 50–10,000 bps (Fig. 5), where the marker lane was loaded with 10 µl while each sample lane was loaded with 15 µl of DNA amplicon.

**Fig. 6:**
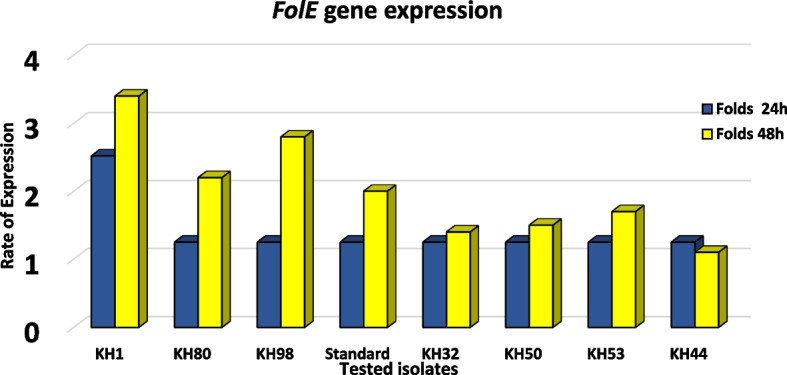
Folds of FolE gene expression in seven selected isolates against standard strain along 48 h of incubation

Using the *RecA* gene as a reference gene, RT-PCR was used to assess the expression of the *folE* gene, which codes for GTP cyclohydrolase I (GCYH-I), a first enzyme in the de novo tetrahydrofolate biosynthetic pathway found in bacteria, fungi, and plants. The expression of the *folE* gene was higher in just one isolate (KH1) with folds of 3 and 3.4 after 24 and 48 h of incubation while standard strain and all others were with onefold only after 24 h of incubation. After 48 h of incubation, *FolE* expression increased in all isolates besides the standard one, within 48 h of incubation. KH98 was the best second folate expression after KH1 with 2.8 folds followed by the KH80 with 2.2 folds, then standard with 2 folds (Fig. 6).

### Growth optimization of the best isolates

#### ***CO***_***2***_*** concentration***

**Fig. 7:**
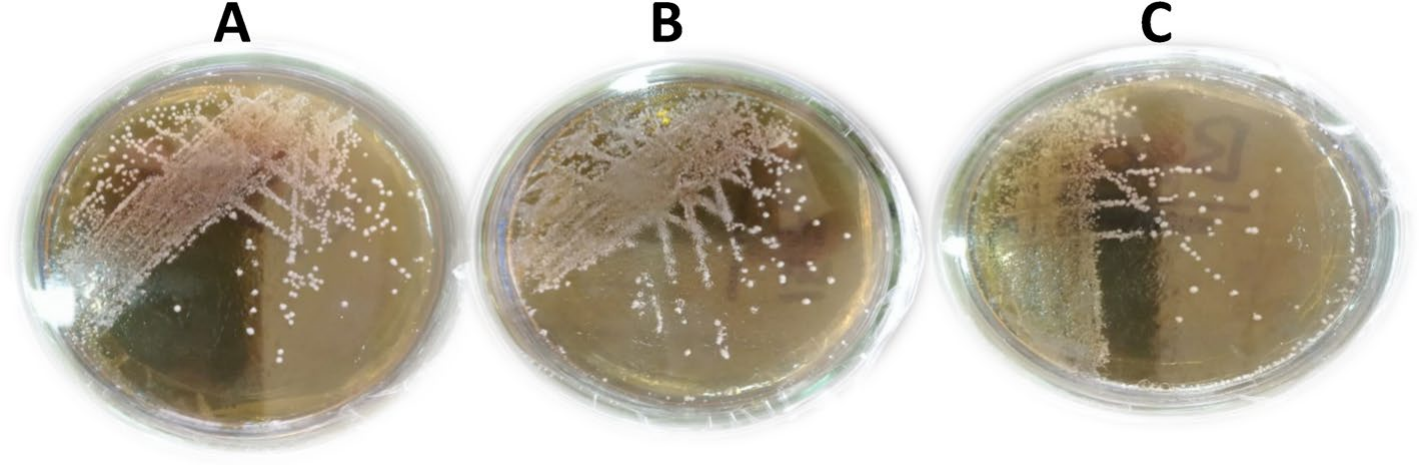
The best folic acid-producing three isolates as grown on MRS agar plates. **A** KH1, **B **KH80, and **C** KH98

The best three folic acid producers and their growth were optimized for many factors; they have grown well at 30°C on MRS agar plate (Fig. 7).

**Fig. 8:**
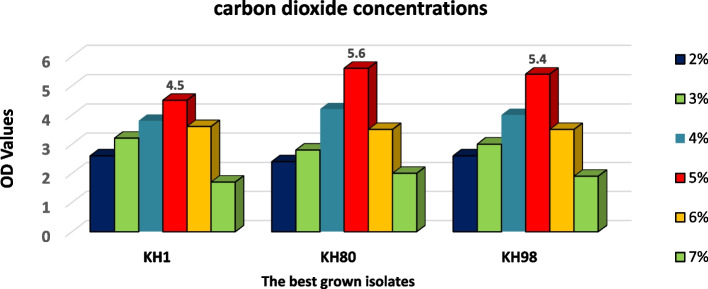
The best three Lactobacilli isolates growth at different CO2 concentrations; 5% CO2 was the best one for almost Lactobacilli growth

Co_2_ concentration is a fundamental factor for lactobacilli growth, so all selected isolates were tested at six different CO_2_ concentrations (2, 3, 4, 5, 6, and 7%). All isolates were grown rapidly and intensively at CO_2_ concentration 5%, and this concentration regarded the optimal concentration for *Lactobacilli* isolates in this study (Fig. 8); the best three growing isolates were KH1, KH80, and KH98.

#### Incubation time

**Fig. 9:**
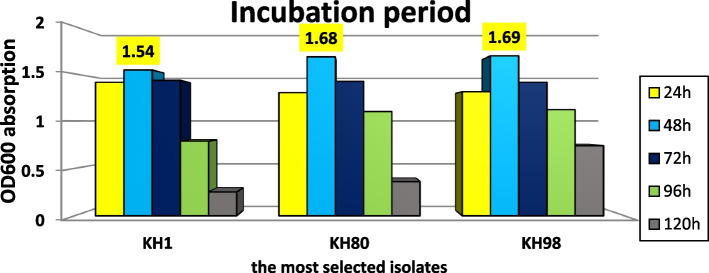
Growth pattern of three isolates as measured calorimetrically at OD600 for determining the exponential phase

The logarithmic growth curve of microorganisms was affected by the time of incubation; at different incubation time intervals, OD600 reads were recorded against the starter (zero time of incubation). The maximum *Lactobacilli* growth was between 24 and 48 h of incubation that suggested to be logarithmic growth phase. All selected isolates showed retardation in growth after 48 h of incubation. Stationary phase was achieved through constant reading at OD600 (Fig. 9). This colorimetric reading was confirmed by counting CFUs on MRS agar each day with triplicate plate for each isolate that was recorded in Table 3.

**Table 3:** Log CFU count of three *Lactobacilli* isolates on MRS agar for 5 days of incubation at 28°C and CO_2_ concentration 5%

	Incubation time/hours				
Isolates	24 h	48 h	72 h	96 h	120 h
KH1	320	360	310	245	210
KH80	245	303	241	217	122
KH98	265	278	290	236	190

CFU count of the most potent three LAB isolates for 5 days

### Optimization of probiotic characters

#### pH range tolerance

Three isolates were tested against wide pH range for determining low pH tolerance as gastric juice range was pH 1–2.5. KH1, KH80, and KH98 highly showed tolerance against extremely acidic pH 2–3; they give 45, 10, and 22 CFUs at pH 3, besides 9, 6, and 4 CFUs at pH 2 respectively and as mentioned in Table 4.

**Table 4:** CFUs counting of seven lactic acid bacterial isolates at wide pH range for 24 h on MRS plates against control (C)

Isolates	pH range							**C**
	8	7	6	5	4	3	2	
KH1	123	145	112	82	77	45	9	147
KH80	120	142	111	45	21	10	6	140
KH98	121	162	98	67	41	22	4	166

Values described in this table were the count of CFUs/ml fermentation after 24 h of incubation in brain heart fusion liquid at optimal carbon concentration for each isolate besides the best CO2 concentration. Values above multiply in dilution factor 10^5^

### Bile salt tolerance

**Fig. 10:**
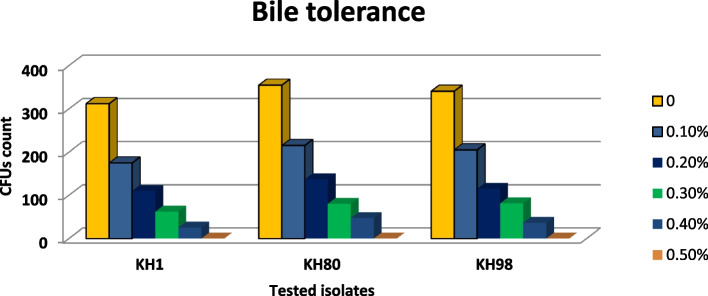
CFUs counting of the selected three isolates at different bile salt concentrations

Among the seven isolates, there were three which showed high resistance against elevated bile salt concentrations when compared with the remaining four; these were KH1, KH2, and KH3 which give CFUs count at 0.3% bile salt 62, 80, and 81. These also give 25, 47, and 36 CFUs with 0.4% bile salt concentration respectively (Fig. 10).

### PCR identification of Lactobacilli through 16s rDNA

**Fig. 11:**
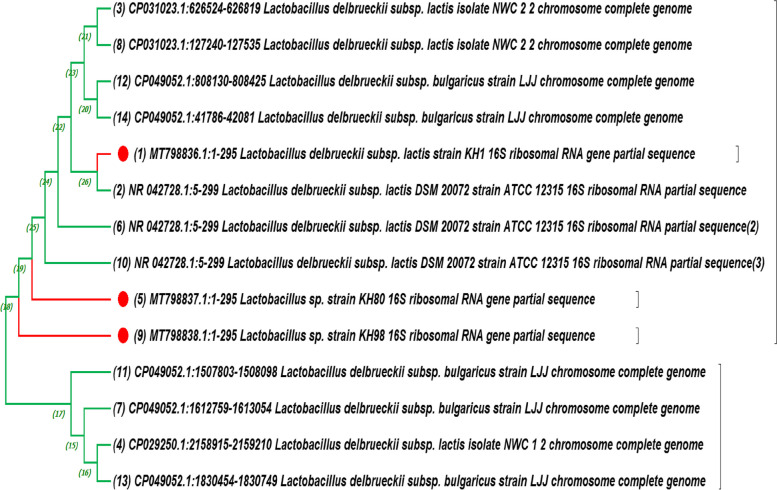
Phylogenetic tree of the selected three isolates as identified Lactobacillus delbrueckii with the highly similar 16S rDNA sequences on NCBI GenBank

The confirmed identification of *Lactobacilli* isolates (the best three with antibacterial activity) carried through amplification of 16s rDNA through PCR reaction using two universal bacterial primers mentioned in Table 1. PCR amplicon of the three isolates was sequenced and analyzed through BLAST on NCBI site for closely related sequences on Gene Bank. These isolates KH1, KH80, and KH98 have accession numbers as follows: MT798836, MT798837, and MT798838, respectively. Phylogenetic trees for the three isolates with the most related strains on NCBI were described in Fig. 11.

**Fig. 12:**
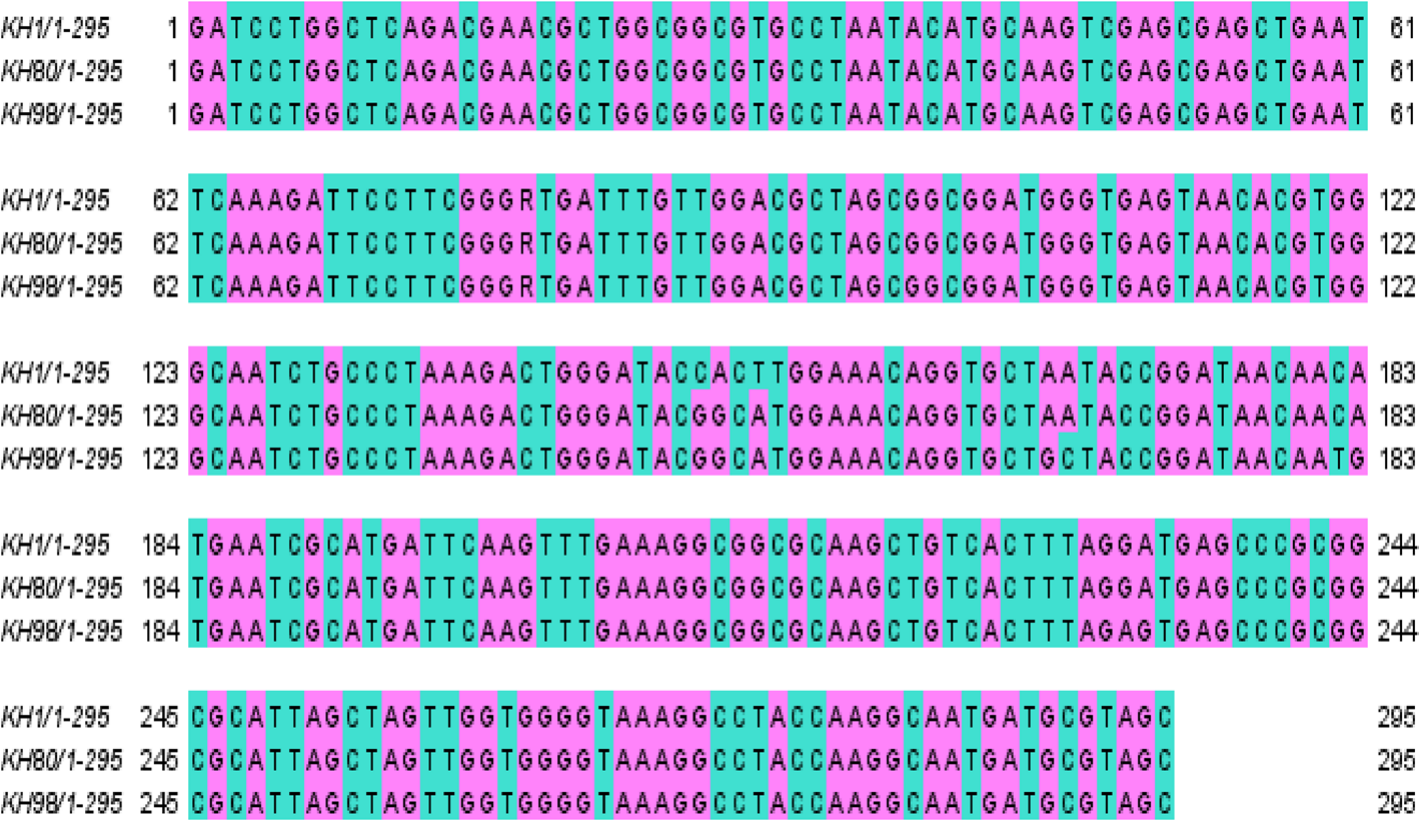
Jalview alignment for three DNA nucleotide sequences KH1, KH80, and KH98 with length 265 nucleotides

Three nucleotide sequences of the three isolates were compared together for similarity percentage through Jalview windows software edition 2.11.1.6. Alignment as in Fig. 12**.**

Single nucleotide polymorphism alignment shows similarity percentage of 98.56 with score 29,020.0 between KH1 and KH80, between KH1 and KH98 similarity percentage of 96.64 with score 27,960.0, and finally between KH80 and KH98 similarity percentage of 97.97 with score 28,440.0.

### Antioxidant assay of KH1 fermentation

**Fig. 13:**
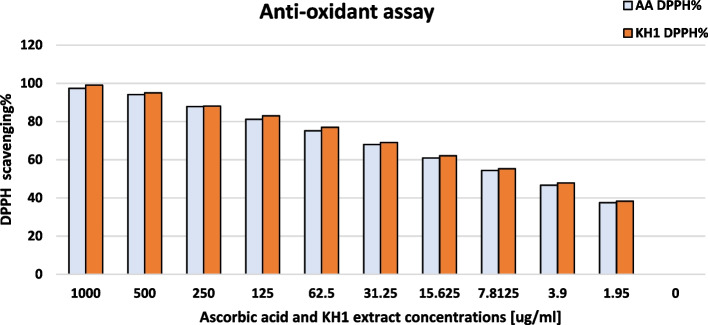
Antioxidant activity of KH1 fermentation against several known concentrations of ascorbic acid through DPPH

Serial dilutions of KH1 fermentation broth were carried out and tested for their antioxidant activity against different known concentrations of ascorbic acid (1.95, 3.9, 7.8, 15.6, 31.2, 62.5, 125, 250, 500, and 1000 ug/ml) through DPPH assay. All dilutions of KH1 fermentations showed higher scavenging percentage than all ascorbic acid concentrations. The highest ascorbic acid concentration of 1000 µg/ml showed 97.3% scavenging while KH1 recorded 99% as described in Fig. 13.

### Docking process

**Fig. 14:**
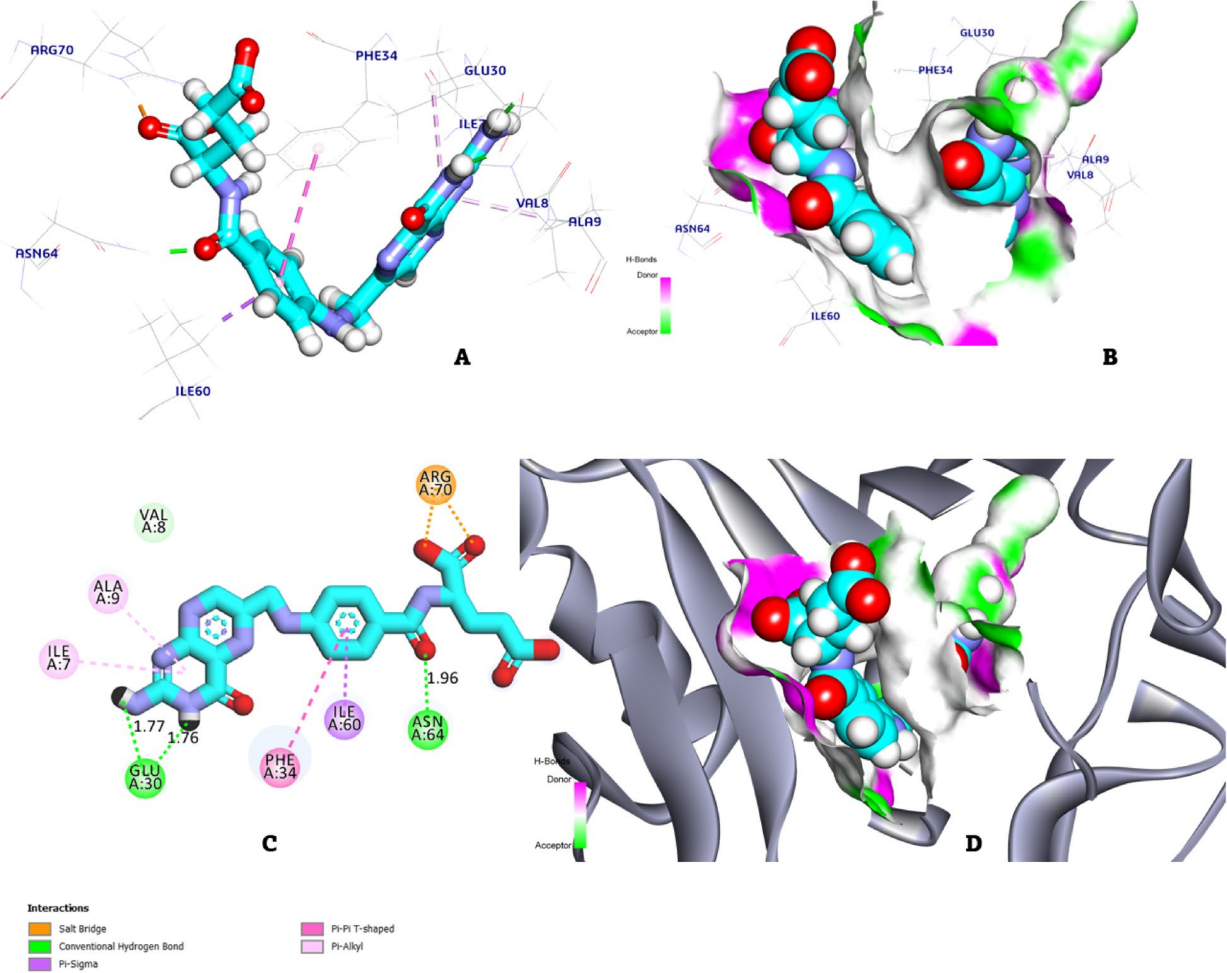
Folic acid docked in DHFR, hydrogen bonds shown in green lines, and the pi interactions are represented in pink lines (Fig. **A**, **C**) with surface mapping showing folic acid occupying the active pocket of DHFR (Fig. **B**, **D**)

Molecular docking was carried out using docking algorithms. The targeted pocket was fixed rigidly while the ligands were moved freely. During the refinement, each molecule was allowed to produce 20 different interaction poses with the protein. Then the docking score (affinity interaction energy) of the best-fitted poses with the active site at DHFR was recorded and 3D orientation was generated by Discovery Studio visualizer software. The binding mode of folic acid exhibited an energy binding of − 8.65 kcal/mol against DHFR. Folic acid formed four Pi-alkyl, Pi-Pi, and Pi-sigma interactions with Ala9, Ile7, Phe34, and Ile60. Additionally, folic acid interacted with Glu30 and Asn64 by three hydrogen bonds with a distance of 1.77, 1.76, and 1.96 Å as shown in Fig. 14.

## Discussion

After incubation on MRS plates, CFUs were counted and examined for their gram stain reaction and morphological examination of colonies; about 100 CFUs reacted positively with gram reaction and appeared in bacillary form under light microscope. Seven bacterial strains among 25 isolates were identified as *Lactobacilli* [24].

Lactic acid bacteria require rich, complex cultivation media for normal growth. The homofermentative LAB converted glucose to lactic acid while heterofermentative LAB fermented glucose to lactic acid, ethanol, acetic acid, and CO2. Simple carbohydrates or sugars are the carbon and energy sources for the growth of LAB. *Lactobacillus spp*. have the ability to convert various types of sugar into lactic acid [25]. Glucose is the most used sugar while lactose is utilized in dairy products by *Lactobacillus* strains [26, 27] reported that besides glucose, maltose, mannose, and galactose are also found to be preferred carbon sources for some LAB. [28] has 2% tryptone and pH 6.5 have been reported to increase antimicrobial activity. *Lactobacillus durans* E204 activity was compared to the control.

Folic acid is generally found in all types of food such as vegetables, fruit, and meat which is an important substance needed for nucleic acid synthesis and cell differentiation. Folic acid influences the physiological development of the fetal nervous system [29] reported that fermented milk bio-enriched with folate was able to increase folate concentrations in plasma and decrease homocysteine levels. Different researchers have reported that LAB could synthesize folate during fermentation depending on the growth conditions [30]. Many researchers report that LAB isolated from various sources such as dairy products, vegetables, and traditional fermented foods could produce folate both extracellularly and intracellularly with different concentration when growing in folate free culture medium [30]. These results were supported by [31] when found that *Lactobacillus delbrueckii subsp.* bulgaricus and *Streptococcus thermophilus* produced highest extracellular folate levels.

After 48 h of incubation, *FolE* expression increased in all isolates besides the standard one, within 48 h of incubation; KH98 was the best second folate expression after KH1 with 2.8 folds followed by the KH80 with 2.2 folds, then the standard with 2 folds. These folds reflect the high folic acid productivity of these isolates [32]. 

Lactic acid bacteria (LAB) use carbohydrates as the main carbon source [32]. The amount of CO2 produced by LAB during the fermentation of sugars depends on several factors, such as type of fermented sugar where fermentation of glucose is faster than lactose, even in the case of active lactose fermenters. Also, the optimum concentration of sugar being about 5% and optimum initial reaction of the medium is approximately pH 7 [33]. A lower concentration of carbon dioxide can stimulate the growth of some microorganisms, but a higher concentration of carbon dioxide can prevent the growth of many spoilage microorganisms, including bacteria and fungi; therefore, it is extensively used in food preservation [34].

Cultivation of lactic acid bacteria is recommended to be under anaerobic conditions by direct incubation in a CO2 incubator at 27 ± 1°C for 5–7 days. A microbial incubation period curve can provide helpful information for understanding microbial growth trends and selecting the optimal growth stage [35].

Yang et al. [36] found that *Lactobacillus spp.* strains isolated from soybeans showed logarithmic growth period between 2 and 8 h. Also, Wu et al. [37] reported that *L. delbrueckii ssp*. has short latency phase of WDS-7 strain and the culture pH value reached to 3.8 after 20 h of incubation; this strain had strong capacity for acid production.

Khalil et al. [33] reported temperature and humidity optimization. pH conditions for secondary metabolite production using pH 8 and 37°C for *Lactobacillus faecium* B3L3 Table 5.

**Table 5:** DG, RMSD, and interactions kcal/mol of folic acid against *targeted sites of* DHFR

Targets screened	Tested compounds	RMSD value (Å)	Docking (affinity) score (kcal/mol)	Interactions	
				H.B interaction	Pi -
DHFR	Folic acid	1.14	− 8.65	3	4

According to Das et al. [38], acid and bile salts around 0.3% tolerance is one of the important criteria for the selection of probiotic strain. The viability value and resistant mechanism towards low pH or bile salts concentration depend on the type and species of bacteria that have ability to survive at a low pH in stimulated gastric juice [23, 39, 40]. Recently, Hsu et al. [41] have found that intestinal probiotics, such as *Lactobacillus* spp., are resistant to the high concentration of bile salts at low pH. Laino et al. [29] stated that *Lactobacillus delbrueckii* ssp. isolated from Qarish cheese showed high tolerance to low pH and could survive under acidic environment of stomach.

Probiotics have numerous health benefits such as antimicrobial and antioxidant activities [42]. Antioxidant activity of probiotics could help the host to destroy and neutralize free radicals [43]. The antioxidant activity of LAB depends on the source and strain. Reactive oxygen species (ROS) is a type of highly active oxygen free radicals which includes superoxide anion radicals, hydrogen peroxide, and hydroxyl radicals [44]. Feng and Wang [45] reported that a small amount of ROS is required for many different cellular activities. Many researches tried to find natural and safer antioxidants from natural resources [46]. The antioxidant activities of *Lactobacillus* spp. strains isolated from pickled tea and traditional fermented foods reached almost from 30 to 80% [38, 47]. Molecular docking studies is a powerful approach for structure-based drug discovery. It can be used for modeling the interaction between a protein at level of atoms besides a tiny molecule. This technique facilitates characterization of small molecules behavior in the binding site of target proteins and to elucidate vital biochemical processes [48].

Firstly, the structures of the folic acid were drawn by DFT program. Then, download the structure of the receptor DHFR form (Ala9, Ile7, Phe34, and Ile60) from protein data bank for use in Auto Dock Tools to identify the active sites of binding mode between the compound and the receptor. The data from the docking studied as receptor, folic acid moiety, receptor site, and binding energies are shown in Table 2. The 3D plots of the interaction of the prepared compounds with receptor were also symbolized in Fig. 12 [49].

Secondly, Folic acid’s binding mechanism exhibits an energy binding of − 8.65 kcal/mol against DHFR. Ala9, Ile7, Phe34, and Ile60 produced four Pi-alkyl, Pi-sigma, and Pi-Pi interactions with folic acid. The binding energy of folic acid to DHFR is higher than chalcone derivatives which ranged from − 6.79 to − 8.02 kcal/mol [50], trimethoprim has a band energy of − 7.09 kcal/mol [50], dihydropyrimidine derivatives have a band energy from − 6.549 to − 8.392 kcal/mol [51], and approximately to the methotrexate which has a binding energy of − 8.78 kcal/mol [52]. Moreover, as seen in Fig. 9, folic acid connected with Glu30 and Asn64 by three hydrogen bonds that had 1.77, 1.76, and 1.96 A^o^.

## Conclusion

LAB Egyptian local isolates exhibit highest folic acid productivity reached 100 µg/ml after 48 h of incubation through KH1 which also with the highest *FoLE* gene expression after 24 and 48 h of fermentation in comparison of standard strain (*Lactobacillus delbrueckii* ATCC 730); KH1 which were identified through 16S rDNA belong to *Lactobacillus delbrueckii* with genbank accession number (MT&98,836.1). All dilutions of KH1 have antioxidant activity with higher scavenging percentage than all ascorbic acid concentrations. Folic acid exhibited an energy binding of − 8.65 kcal/ mol against DHFR. Folic acid formed four Pi-alkyl, Pi-Pi, and Pi-sigma interactions with Ala9, Ile7, Phe34, and Ile60. Additionally, folic acid interacted with Glu30 and Asn64 by three hydrogen bonds with 1.77, 1.76, and 1.96 Å. 

## Data Availability

All data included in this study were presented in the form of tables and figures.
